# Fighting flu: novel CD8^+^ T‐cell targets are required for future influenza vaccines

**DOI:** 10.1002/cti2.1491

**Published:** 2024-02-14

**Authors:** Samuel Liwei Leong, Stephanie Gras, Emma J Grant

**Affiliations:** ^1^ Department of Biochemistry and Chemistry, La Trobe Institute for Molecular Science La Trobe University Bundoora VIC Australia; ^2^ Department of Biochemistry and Molecular Biology, Biomedicine Discovery Institute Monash University Clayton VIC Australia

**Keywords:** CD8^+^ T cells, epitopes, HLA‐I, influenza, vaccines

## Abstract

Seasonal influenza viruses continue to cause severe medical and financial complications annually. Although there are many licenced influenza vaccines, there are billions of cases of influenza infection every year, resulting in the death of over half a million individuals. Furthermore, these figures can rise in the event of a pandemic, as seen throughout history, like the 1918 Spanish influenza pandemic (50 million deaths) and the 1968 Hong Kong influenza pandemic (~4 million deaths). In this review, we have summarised many of the currently licenced influenza vaccines available across the world and current vaccines in clinical trials. We then briefly discuss the important role of CD8^+^ T cells during influenza infection and why future influenza vaccines should consider targeting CD8^+^ T cells. Finally, we assess the current landscape of known immunogenic CD8^+^ T‐cell epitopes and highlight the knowledge gaps required to be filled for the design of rational future influenza vaccines that incorporate CD8^+^ T cells.

## Graphical abstract

Seasonal influenza viruses continue to cause severe medical and financial complications annually despite vaccines being available. As such, there is a need for updated vaccines that provide long‐lasting protection to global populations. In this review, we summarise licenced influenza vaccines and those currently in clinical trials, highlighting their key advantages and limitations. Furthermore, we discuss activating CD8^+^ T cells for future influenza vaccinations, highlighting the current gaps in knowledge for rational vaccine design.

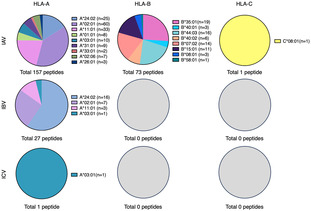

## Influenza viruses

In the 21st century, just over 100 years since the outbreak of the pandemic A/H1N1 influenza virus which caused the devastating 1918–1919 Spanish Flu pandemic, influenza remains an ongoing threat to humans.^1^ Epidemiological studies from the World Health Organization (WHO) estimate that roughly 650 000 deaths are attributed to seasonal influenza infections annually.^2^ Influenza viruses are single‐stranded RNA viruses with segmented genomes which encode for a range of structural and non‐structural proteins. There are several influenza virus subtypes, three of which are known to infect humans, the influenza A virus (IAV), the influenza B virus (IBV) and the influenza C virus (ICV).^3^

Generally, influenza symptoms may vary from mild to severe, requiring hospitalisation.^4^ Mild cases of influenza are typically present with cough, feverish symptoms, diaphoresis (cold sweats) and headaches.^4^ Conversely, severe cases of influenza can lead to acute respiratory distress syndrome (ARDS).^5^ ARDS is described as a hyperactive immune reaction resulting in severe damage to the lungs, without neutralisation of the virus.^5^ This leads to pulmonary oedema, organ failure and eventually death, if left untreated.^5^

Of the influenza viruses that circulate and infect humans, IAV is the most characterised and is the only influenza virus that has caused a pandemic so far. The IAV contains eight gene segments known as polymerase basic proteins 1 and 2 (PB1 and PB2), non‐structural protein (NS), nucleoprotein (NP), the matrix protein (M), polymerase acidic protein (PA), haemagglutinin (HA) and neuraminidase (NA).^6^, ^7^ These genes encode for 18 known proteins, of which 10 are functionally critical (PB1, PB2, PA, NP, HA, M1, M2, NA, NS1 and NS2).^7^ The nomenclature of IAVs is characterised by the expression of surface glycoproteins, haemagglutinin and neuraminidase.^8^, ^9^, ^10^ For example, the A/H3N2 IAV strain expresses the HA subtype 3 and NA subtype 2.^8^ Currently, 18 different HA and 11 NA subtypes have been described. Uniquely, IAV can also infect several animals (e.g. birds, pigs and horses), and phylogenetic evidence suggests that all mammal‐derived IAVs are derived initially from avian sources.^11^ The ability to infect several animal species creates a viral reservoir that can subsequently infect humans, making it fundamentally impossible to eradicate.^8^, ^12^

Influenza B viruses primarily contribute to seasonal infections only and are the leading cause of influenza virus infection every few years.^13^ Influenza B viruses are differentiated by their lineages, namely the Yamagata and Victoria lineages.^14^ IBVs correspondingly spread between humans and are typically not known to infect any animal reservoirs.^15^ The IBVs are also known to share the same genes and proteins as IAVs.^16^

The ICV typically affects younger children and is thought to be highly underreported as it typically induces a mild disease which can be overlooked as various other respiratory illnesses.^17^ According to some reports, it has been stated that many young children will be infected with the ICV at some point during their childhood.^17^ What distinguishes the ICV from the other family of influenza viruses is that they have one less gene segment (totalling seven gene segments).^17^ Furthermore, ICV also encodes for the haemagglutinin esterase fusion glycoprotein which is important for viral entry.^17^

Typically, there are four influenza virus strains (2 IAV and 2 IBV) that continually and simultaneously circulate between hemispheres within humans that contribute to seasonal infections.^2^ These are the A/H1N1 and A/H3N2 strains of influenza A, and the Yamagata and Victoria lineages of influenza B.^18^ Moreover these are the targets of the majority of the licenced vaccines administered around the world.^18^ The ICV is not a priority of the WHO and thus is not a target of influenza vaccines.^2^, ^19^

One important characteristic of all influenza viruses is that they lack the molecular mechanisms to proofread genomic RNA, which can lead to point mutations.^20^ These point mutations result in antigenic drift, which can decrease protection from pre‐existing immunity and is typically responsible for seasonal epidemics. This is the catalyst for annual influenza vaccine updates.^20^ This occurs particularly with IAV, as the IAV can circulate in many animal reservoirs, under unique circumstances the IAV can undergo antigenic shift.^21^, ^22^ Antigenic shift generally occurs when two unique IAV strains co‐infect a host, and through genetic reassortment a new strain emerges.^22^, ^23^ If the new strain is significantly different to previously circulating strains, there is a potential to evade pre‐existing immunity.^23^ If the reassorted virus is efficient in replication and transmission, it may also result in a pandemic.^23^ Thus, the total expected mortalities can significantly exceed seasonal influenza epidemics.^23^, ^24^ In other instances, the effect of antigenic shift may contribute to seasonal epidemics, as well as pandemic‐like strains of previous years.^25^, ^26^

So far, vaccines are the most used therapeutic worldwide to prevent severe influenza disease, particularly in vulnerable individuals such as children (< 5 years of age), the elderly (> 65 years of age), pregnant women, the immunosuppressed and individuals with co‐morbidities.^27^ While antivirals are used is select circumstances, influenza infections continue to cause significant morbidity and mortality annually.^27^, ^28^, ^29^ According to the Centers for Disease Control and Prevention (CDC), the 2019–2020 season had a high‐end estimate of more than 30 million symptomatic cases (~9% of the US population as of 2023) reported in the United States.^30^, ^31^ It has been estimated that roughly 50 million individuals will be infected with the influenza virus (~14.7% of the US population) and more than 650 000 hospitalisations will occur in the 2022–2023 season.^32^

Apart from morbidity and mortality, influenza virus infections also cause a significant financial liability.^33^ In a recent systematic review, Courville *et al*. categorised the cost of influenza burden into two groups: direct and indirect costs. Direct cost encompasses the medical intervention of outpatient and inpatient expenses, while indirect costs are dependent on approved sick leave and workplace inefficiency caused by sicknesses.^33^ Unsurprisingly, this study observed that the annual indirect cost of influenza amounted to roughly US$8 billion in the United States alone.^33^ Thus, new and broadly protective influenza vaccines are a topic of great interest and research.

## Current influenza vaccines

When considering the development of novel influenza virus vaccines of the future, it is important to understand the advantages and disadvantages of current vaccines for the generation of novel therapeutics. Currently there are 19 influenza virus vaccines (not including variations of same vaccine) licenced for use by the Food and Drug Administration (FDA) (USA), the Therapeutics Goods Administration (TGA) (Australia) and the European Medicines Agency (Europe) (Table 1). The available vaccines include subunit vaccines, live attenuated vaccines and surface protein inactivated vaccines (Table 1). Influenza vaccines are typically designed to simultaneously protect against two antigenic strains of IAV and one (trivalent vaccines) or two (quadrivalent vaccines) lineages from IBV.^34^, ^35^ Most of these vaccines generally induce a strong neutralising antibody (nAb) response against the surface HA glycoprotein.^35^, ^36^ The advantage of inducing a strong nAb response via vaccination is to neutralise the virus before its entry into host cells, thereby preventing infection and viral replication.^37^ It is important to note that these vaccines may induce other non‐neutralising antibodies with antibody‐dependent cellular cytotoxicity (ADCC), complement‐dependent cytotoxicity or dependent cell‐mediated phagocytosis.^38^ However, nAbs are traditionally measured to assess vaccine efficacy, as they are considered the ‘correlate of protection’. Interestingly, there are now several additional proposed ‘correlates of protection’ that, once verified, could be used to assess the efficacy of future influenza vaccines.^39^ Importantly, antibody‐based vaccines are very well characterised, generally well tolerated and therefore deemed safe.^37^

**Table 1:** Currently licenced influenza vaccines

Name of vaccine	Type of vaccine	Protein immunogen	Manufacturer	Targeted population	Authorised use (country/union)
Vaxigrip Tetra	Surface antigen inactivated influenza vaccine (egg‐based)	4× HA	Sanofi‐Aventis (Australia)	≥ 6 months	TGA
Fluquadri	Surface antigen inactivated influenza vaccine (egg‐based)	4× HA	Sanofi‐Aventis	≥ 6 months	TGA
Fluzone (quadrivalent), Fluzone high‐dose (quadrivalent) and Fluzone intradermal (quadrivalent)	Split virion/inactivated influenza vaccine (egg‐based)	4× HA	Sanofi Pasteur (USA)	≥ 6 months (Fluzone quadrivalent USA) ≥ 60 years (Fluzone high‐dose Australia) ≥ 65 years (Fluzone high‐dose USA) 18–64 years (Fluzone intradermal USA)	FDA TGA
Fluzone (trivalent), Fluzone intradermal (trivalent), Fluzone high‐dose (trivalent)	Split virion/inactivated influenza vaccine (egg‐based)	3× HA	Sanofi Pasteur (USA)	≥ 6 months (Fluzone quadrivalent USA) 18–64 years (Fluzone Intradermal USA) ≥ 65 years (Fluzone high‐dose USA)	FDA
Fluarix Quad and Fluarix (trivalent)	Split virion/inactivated influenza vaccine (egg‐based)	3× or 4× HA	GlaxoSmithKline Biologicals (USA)	≥ 6 months	FDA
Fluarix Tetra	Split virion/inactivated influenza vaccine (egg‐based)	4× HA	GlaxoSmithKline	≥ 6 months	TGA
Flublok quadrivalent and Flublok trivalent	Recombinant influenza vaccine	3× or 4× HA	Protein Sciences Corporation	≥ 18 years	FDA
Flulaval quadrivalent and Flulaval (trivalent)	Split virion/inactivated influenza vaccine (egg‐based)	3× or 4× HA	ID Biomedical Corporation of Quebec	≥ 6 months	FDA
Fluad quadrivalent and Fluad (trivalent)	Surface antigen/inactivated influenza vaccine (egg‐based)	3× or 4× HA	Seqirus	≥ 65 years (Fluad quadrivalent Australia) ≥ 65 years (Fluad quadrivalent and trivalent USA)	TGA FDA
Fluad Tetra	Surface antigen/inactivated influenza vaccine (egg‐based)	4× HA + NA (only HA quantity reported)	Seqirus, the Netherlands	≥ 65 years	European Medicines Agency
Flumist Quad and Flumist	Live attenuated vaccine	Various influenza proteins	MedImmune, LLC	2–49 years	FDA
Afluria Quad and Afluria Trivalent	Split virion/inactivated influenza vaccine (egg‐based)	4× HA	Seqirus (Australia and USA)	≥ 5 years (Alfuria quad Australia) ≥ 18 years (Alfuria quad and trivalent USA)	TGA FDA
Flucelvax quad and Flucelvax trivalent	Surface antigen/inactivated influenza vaccine (cell‐based)	3× or 4× HA	Seqirus	≥ 2 years (Flucelvax quad Australia) ≥ 6 months Flucelvax quad and trivalent (USA)	TGA FDA
Flucelvax Tetra	Surface antigen/inactivated influenza vaccine (cell‐based)	4× HA + NA (only HA quantity reported)	Seqirus	≥ 2 years	European Medicines Agency
Influvac Tetra	Surface antigen/inactivated influenza vaccine (egg‐based)	4× HA	Viatris (Australia)	≥ 6 months (Australia)	TGA
Fluenz Tetra	Live attenuated vaccine	Various influenza proteins	AstraZeneca	2–18 years	European Medicines Agency
Agriflu	Surface antigen/inactivated influenza vaccine (egg‐based)	3× HA + NA (only HA quantity reported)	Seqirus	≥ 18 years	FDA
Supemtek	Recombinant influenza vaccine (cell‐based)	4× HA	Sanofi Pasteur	≥ 18 years	European Medicines Agency
Fluvirin	Subunit/inactivated influenza vaccine (egg‐based)	4× HA + NA (only HA quantity reported)	Seqirus	≥ 4 years	FDA

Food and Drug Administration, Therapeutic and Goods Administration and European Medicines Agency approved influenza vaccines. Lists of approved vaccines were gathered from their respective governmental administrative databases (February 2023) including FDA at https://www.fda.gov/vaccines‐blood‐biologics/vaccines/vaccines‐licensed‐use‐united‐states, TGA at https://www.ebs.tga.gov.au/ebs/picmi/picmirepository.nsf/PICMI?OpenForm&t=PI&q=Afluria&r=/ and European medicines agency vaccines at https://www.ecdc.europa.eu/en/seasonal‐influenza/prevention‐and‐control/vaccines/types‐of‐seasonal‐influenza‐vaccine and https://www.ema.europa.eu/en. Age, composition and target population information was gathered directly from the manufacturer's websites, the Australian Government Australian Immunisation Handbook https://immunisationhandbook.health.gov.au/ and the FDA and European medicines agency websites as earlier.

## Limitation of current influenza vaccines

One major disadvantage of current antibody‐based influenza vaccines is that the majority target the HA viral glycoproteins that are highly susceptible to mutations.^20^ This is a significant issue, as nAbs are unable to neutralise antigenically differentiated strains, and as such, the previous year's vaccine may not provide cross‐protection to current circulating strains.^37^ For this reason, influenza vaccines require yearly reformulations as per the WHO's recommendations.^2^

Another significant disadvantage of current influenza vaccines is the lengthy manufacturing process. Influenza virus proteins are typically grown in embryonated hens' eggs which can take in excess of 6 months, and therefore relies on prediction of upcoming antigenic strains more than 6 months into the future.^40^ Moreover, the use of chicken eggs may also not be suitable for all individuals.^41^ For example, egg allergies are the second most common leading food allergy besides milk in children and may pose a problem during vaccinations.^41^, ^42^ Additionally, the WHO gathers information from the WHO Global Influenza Surveillance and Response System (GISRS) that monitors and predicts upcoming antigenic strains by surveying influenza data collected.^2^ With this information, a recommendation is made for the upcoming influenza virus season.^2^ Given predictions are required so far in advance, recommendations occasionally result in a mismatch between the predicted vaccine strain and the actual circulating strain.^43^ This was seen in the 2014–2015 season in the United States which resulted in a 6% overall vaccine efficacy against the A/H3N2 strain.^44^ Furthermore, the growth of influenza viruses have been known to introduce mutations which can lead to further variations in vaccine efficacy.^45^ Even when the predicted strains do match the circulating strains, vaccine efficacy can be highly variable. Several studies that have systematically reviewed databases such as PubMed and Embase,^46^, ^47^ reported an overall vaccine efficacy of 33% against the A/H3N2 influenza strain (pooled) from the years 2004–2015.^46^, ^47^ Although currently licenced influenza vaccines have been effective, novel vaccines are required to counteract the disadvantages of currently licenced vaccines and provide long‐lived, broader protection against distinct influenza virus strains, preventing the need for annual vaccination.

## Novel technologies and upcoming influenza vaccines

The need for an updated influenza vaccine to address the above‐mentioned disadvantages is a topic of high activity in the scientific community, with many new influenza vaccines currently undergoing clinical trials.^48^, ^49^, ^50^ Many of these have been recently reviewed in depth by Hu *et al*.^34^ and as such, we make note the type of vaccine and their key advantages and disadvantageous. Novel technologies for influenza virus vaccines include genetically modified influenza virus vaccines, virus‐like particle (VLP) vaccines, nanoparticle vaccines, viral vector vaccines, mRNA vaccines and recombinant protein‐based vaccines (Table 2).^34^ Interestingly, these novel technologies make use of different molecular mechanisms and different routes of vaccination to deliver the antigen to their target locations within the body, and this has been reviewed by Hu *et al*.^34^

**Table 2:** An overview of selected clinical trial vaccines for influenza

Vaccine technology and example	Typical influenza protein target	Immune‐targeted response	Advantages	Reference
Genetically modified influenza vaccines – RedeeFlu M2SR	Haemagglutinin and neuraminidase	Neutralising antibody response	Stimulate a natural infection with lower risk of adverse events	44
Nanoparticle vaccines – NANOFLU	Haemagglutinin	Neutralising antibody response and CD4^+^ T‐cell activation	Nanoparticles can deliver specific proteins, peptides or mRNA material intracellularly	57
Virus‐like particles – quadrivalent VLP	Haemagglutinin	Neutralising antibody response and CD4^+^ T‐cell activation	Do not contain viral genetic material, can express target proteins and VLP vaccines have been used successfully in the past against HPV	63
Viral vector vaccine – NasoVAx	Haemagglutinin	Neutralising antibody response and T‐cell activation	Can deliver specific genetic influenza genes via an unrelated virus and can stimulate T cells	67
mRNA vaccine – Pfizer‐bionTech	Haemagglutinin	Activation unknown/undisclosed	The technology has been proven to be successful against SARS‐CoV‐2	71, 104
Protein based vaccine – FLU‐V	Matrix 1, matrix 2 and nucleoprotein	Neutralising antibody response, CD4^+^ and CD8^+^ T‐cell activation	Can target more conserved internal proteins that are less likely to be mutated	72

Clinical trial vaccines and their respective examples are listed against the influenza virus. Genetically modified influenza vaccines, nanoparticle vaccine, virus‐like particles, vector‐based vaccines, mRNA‐based vaccine and protein‐based vaccine are listed with their targeted antigen and immune response.

Genetically modified influenza vaccines are analogous to the conventional live attenuated influenza vaccines (LAIV), which are, as their name suggests, live and mimic natural infection, but are designed to have decreased viral virulence to prevent the establishment of disease (i.e. mutated to be replication deficient at body temperature).^51^ Conversely, genetically modified virus vaccines utilise other methods to decrease viral virulence or pathogenicity, namely de‐optimising or removing influenza proteins or generating chimeric influenza strains.^34^, ^51^ These vaccines are typically delivered intramuscularly or intranasally.^34^ An advantage of genetically modified influenza vaccines is that they simulate a natural influenza infection like LAIVs, with reduced risk of adverse reactions.^34^, ^44^, ^52^ The RedeeFlu M2SR vaccine is a genetically modified A/Brisbane/10/2007(H3N2) influenza virus vaccine currently in phase II clinical trials.^44^ The virus used in this vaccine is M2 deficient.^44^ The M2 gene is necessary for viral entry, viral assembly and viral egress and as such, the M2‐deficient virus can only replicate once.^44^, ^53^, ^54^ The RedeeFlu M2SR vaccine displayed promising nAb response efficacy towards the antigenically similar A/Belgium/4217/2015(H3N2) strain, while having minor adverse reactions compared to the placebo.^44^

Nanoparticles are another technology being utilised for influenza vaccine design, acting as a delivery vehicle for proteins, peptides or mRNA. As the name implies, these vaccines contain small particles made up of either organic or inorganic materials.^55^, ^56^ These vaccines are injected intramuscularly and are appealing for vaccine development as they can deliver specific proteins internally via cell‐mediated endocytosis or externally.^55^ NanoFlu is a nanoparticle vehicle vaccine that is currently in phase III clinical trials targeting adults ≥ 65 years.^57^ The vaccine incorporates four separate HA proteins as advised by the WHO (2019–2020 season).^57^ Compared to a conventional quadrivalent inactivated vaccine, NanoFlu showed similar vaccine efficacies (via haemagglutination nAb titre response) and additionally induced a large multifunctional CD4^+^ T‐cell response.^57^ It was noted, however, that it had a higher occurrence of adverse reactions than the control quadrivalent inactivated influenza vaccine.^57^

Virus‐like particles are a specific type of nanoparticle which are also being utilised for influenza vaccines.^34^ VLPs are generated by expressing viral proteins derived from structural proteins.^58^, ^59^ When expressed, a phenomenon occurs where viral proteins fold and form an outer structure of a virus.^58^, ^59^ From there, viral antigens, like HA and NA proteins, can be expressed and bound to the surface of the viral architecture.^58^, ^59^, ^60^ An advantage of VLP is that they do not contain viral genetic material, which makes them relatively safe as they cannot undergo viral replication.^58^ VLP can be administered intramuscularly and have made highly successful vaccines against viruses in the past such as human papillomaviruses (HPV).^61^, ^62^ Against influenza, the quadrivalent VLP vaccine made by Medicago recently underwent phase III clinical trials.^63^ The quadrivalent VLP vaccine is expressed in plants and contains four separate HA proteins (recommended by the WHO), aimed at inducing a nAb and CD4^+^ T‐cell response.^63^ Although deemed safe, unfortunately the vaccine did not meet its primary efficacious endpoint, and a lot‐to‐lot consistency trial commenced, which supported their initial endpoint.^63^, ^64^

Viral vector vaccines comprise whole replicative‐deficient unrelated viruses, typically adenoviruses, as vectors for the delivery through several vaccination routes^65^, ^66^ of specific genetic material (e.g. DNA plasmids, mRNA) and stimulate the production of the viral proteins of interest. An advantage of viral vectors is that they can induce humoral and T‐cell‐mediated immunity.^65^ The NasoVAX vaccine utilises a genetically engineered replication‐deficient vector expressing an intact HA gene from the A/California/04/2009(H1N1)‐like influenza strain.^67^ The completed phase II clinical trial concluded that NasoVAX accomplished a 100% seroprotection rate [a measurement of the proportion of participants with a haemagglutination inhibition (HAI) titre of ≥ 1:40], similar to the registered quadrivalent inactivated control vaccine.^67^ NasoVAX also recorded an ELISPOT assay measuring IFNγ production in T cells which indicated a higher production of cytokines than control.^67^

Influenza vaccines utilising mRNA technology typically encased in a nanoparticle delivery vector are also in clinical trials.^34^ mRNA technology was brought into the public eye by the COVID‐19 pandemic, as many of the vaccines that were available were mRNA based.^68^ mRNA vaccines utilise messenger RNA sequences of the viral protein of interest encapsulated in a lipid nanoparticle (LNP), which when delivered intramuscularly can facilitate viral protein translation.^69^ Vaccines utilising mRNA technology have been successful in combating SARS‐CoV‐2 infection, the causative agent of COVID‐19, and are now being trialled for the influenza virus.^34^, ^70^ Both Moderna and Pfizer‐BioNTech vaccines are using mRNA technology to encode four separate HA glycoproteins and are currently in phase III clinical trials.^34^, ^71^

Finally, protein‐based vaccines are also being used in the fight against influenza viruses.^34^, ^72^ Protein‐based vaccines typically incorporate large peptides or segments of whole protein (typically 20–35 amino acids) that stimulate humoral and cellular‐mediated immunity (CMI) following subcutaneous or intramuscular vaccination.^34^, ^72^ Some examples of protein‐based vaccines that are ongoing in clinical trials are the M‐001 vaccine from BiondVax Pharmaceuticals (Phase III), the FP‐01.1 vaccine from Immune Targeting Systems (Phase I) and the FLU‐v vaccine from ConserV Bioscience (Phase II).^34^ In the case of the M‐001 vaccine, a single protein consisting of B‐cell, CD4^+^ and CD8^+^ T‐cell epitopes from the NP, HA and M1 proteins is expressed in an *Escherichia coli* bacterial system.^73^, ^74^ For FLU‐v, a different approach is used, where four peptides of the conserved M1 (32‐mer), M2 (24‐mer) and NP (flu A: 20‐mer and flu B: 19‐mer) were synthetically generated.^72^ While the FP‐01.1 vaccine incorporates six different 35‐mer peptides from the PB1, PB2, NP and the matrix proteins, which together are joined using fluorocarbons.^75^ What is comparable between the M‐001, FP‐01.1 and FLU‐v vaccines is that they utilise proteins or protein‐derived peptides from proteins more conserved than the surface glycoproteins counterpart.^72^, ^73^, ^75^, ^76^ This is advantageous as the GISRS recommendations are not necessarily required for this vaccine.

Regardless of the different vaccine approaches, further research, clinical trial safety and vaccine efficacy endpoints must be met before the approval of vaccine can be used for administration. Of note, many of these clinical trials evaluate only nAb as a measure of efficacy, without considering other types of immune responses that may be being induced.

## Exploiting CD8^+^ T cells for future influenza virus vaccines

The numerous vaccines that are currently ongoing in clinical trials have many advantages and disadvantages when protecting against future influenza strains. However, it would be of benefit to design and manufacture vaccines that are independent of predictions and can protect against several influenza viral subtypes. Targeting more conserved sections of the influenza virus that can induce CD8^+^ T‐cell responses towards antigenically distinct strains, along with B‐cell and antibody responses, may be one way to achieve this.^21^, ^77^

Following activation by a pathogen‐derived peptide, CD8^+^ T cells can produce cytolytic molecules such as granzymes and perforins that can directly destroy infected cells.^78^, ^79^ Furthermore, CD8^+^ T cells also produce cytokines such as IFNγ and TNF, which recruit neighbouring immune cells to the site of infection to assist in viral clearance.^78^, ^79^, ^80^, ^81^ CD8^+^ T cells have been well studied in the context of influenza virus and are known to be protective,^82^ and this has been recently reviewed in detail.^16^ For example, in 1983, an article published by McMichael *et al*. demonstrated that CD8^+^ T cells played a crucial role in viral clearance and more importantly demonstrated the ability to recognise antigenically distinct strains via cross‐reactivity.^82^ Furthermore, CD8^+^ T cells have also been shown to decrease the severity of influenza disease. Indeed, functional CD8^+^ T cells decreased influenza‐like illnesses in a cohort infected with the A/H1N1‐pdm‐09 virus.^83^ Others showed that individuals with cross‐reactive memory CD8^+^ T cells were observed to recover more swiftly than those lacking memory CD8^+^ T cells.^84^, ^85^ Additionally, memory CD8^+^ T cells specific to influenza are known to be long‐lived and have been identified directly *ex vivo* over a 13‐year time course.^86^ This evidently demonstrates the potential effectiveness of targeting CD8^+^ T cells in future influenza vaccines.^87^, ^88^ Finally, the necessity to provide protection to those in high‐risk populations, such as Indigenous populations, are of high priority, and a CD8^+^ T‐cell mediated vaccine may bridge this gap and thus reduce the severity of disease in these individuals.^89^ According to some reports, Indigenous populations (especially Indigenous Australians) are 6 times more likely to be hospitalised than non‐Indigenous populations and can be attributed to chronic diseases, lack of or limited access to medical service and lower socio‐economic status.^89^, ^90^

## Designing CD8^+^ T‐cell‐based vaccines for broad population coverage: The challenges of HLA‐I polymorphism

Targeting a CD8^+^ T‐cell response in conjunction with B‐cell and antibody responses via vaccination is clearly of benefit, and some of the previously described vaccine technologies are clearly capable of inducing these responses. CD8^+^ T cells recognise short peptides, typically 8–10 amino acids long, that are presented by the human leukocyte antigens class I (HLA‐I).^91^ These HLA‐I molecules are genetically encoded and are highly polymorphic,^92^ and it is a significant challenge when selecting influenza derived targets that can induce CD8^+^ T‐cell responses (epitopes) and also provide protection to the global population.^93^ More than 25 000 distinctive HLA‐I alleles have been identified to date.^94^, ^95^, ^96^ Moreover, HLA‐I molecules have distinct motif preferences for peptides [preferences based on different residues at position 2 (P2) and the last position (PΩ)].^97^, ^98^ Interestingly, several HLA‐I are grouped into superfamilies based on shared peptide motif preferences.^97^ The polymorphism and peptide motif preferences of HLA‐I presents a profound challenge in selecting the targets to be included in a CD8^+^ T‐cell‐mediated vaccine.^94^, ^96^, ^99^ Furthermore, because of the genetic nature of inheritance, distinct HLA profiles can be linked to certain geographical locations and ethnicities.^21^, ^92^, ^95^ Despite this apparent bottleneck,^21^, ^94^, ^95^ including within a vaccine, several immunogenic CD8^+^ T‐cell targets presented by prevalent HLA‐I molecules, and perhaps even multiple HLA‐I molecules in the same superfamily, may provide a solution to provide broad population coverage for a future T‐cell‐mediated vaccine that could limit viral escape and provide long‐term protection.

## Many highly prevalent HLA‐I molecules have no known influenza‐derived epitopes

Although there have been many studies identifying and characterising immunogenic influenza‐derived peptides restricted to different HLA molecules,^3^, ^16^, ^85^, ^87^, ^100^, ^101^, ^102^ we are not aware of any published systematic review of known immunogenic epitopes restricted to the most prevalent HLA‐I molecules expressed worldwide. Using the database created by Solberg *et al*., we noted the top 10 most prevalent HLA‐A, ‐B and ‐C molecules expressed worldwide (Table 3). Interestingly, the cumulative frequency (which does not account for the co‐expression of HLA‐I molecules within a single individual) of the top 10 HLA‐A, HLA‐B and HLA‐C molecules exceeds 100% population coverage. This suggests that including a single conserved and immunogenic epitope (*n* = 30 epitopes) or a segment(s) of protein(s) containing multiple immunogenic epitopes could provide significant CD8^+^ T‐cell‐mediated protection via vaccination for most if not all individuals worldwide (Table 3).

**Table 3:** Top 10 HLA‐A, HLA‐B and HLA‐C alleles and their global prevalence

HLA prevalence	HLA‐A		HLA‐B		HLA‐C	
	Allele	Frequency (%)	Allele	Frequency (%)	Allele	Frequency (%)
1	A*24:02	18.82	B*35:01	5.47	C*07:02	13.10
2	A*02:01	15.28	B*51:01	5.22	C*04:01	11.18
3	A*11:01	11.66	B*40:01	5.12	C*03:04	9.13
4	A*01:01	4.84	B*44:03	4.47	C*01:02	8.48
5	A*03:01	4.27	B*40:02	4.18	C*07:01	6.89
6	A*31:01	4.09	B*07:02	4.11	C*06:02	6.16
7	A*33:03	4.08	B*15:01	3.43	C*03:03	5.58
8	A*02:06	3.47	B*07:04	3.12	C*08:01	4.52
9	A*26:01	3.35	B*08:01	2.96	C*15:02	3.36
10	A*30:01	2.51	B*58:01	2.89	C*12:02	3.19

The top 10 HLA‐A, B and C alleles were acquired in February 2023. The global frequency of each HLA‐A, B and C alleles were obtained from the http://pypop.org/popdata/2008/byfreq‐A.php.html database.^95^ Each HLA allele is ranked from 1 to 10, with 1 being the highest global frequency and 10 the lowest frequency.

**Figure 1:**
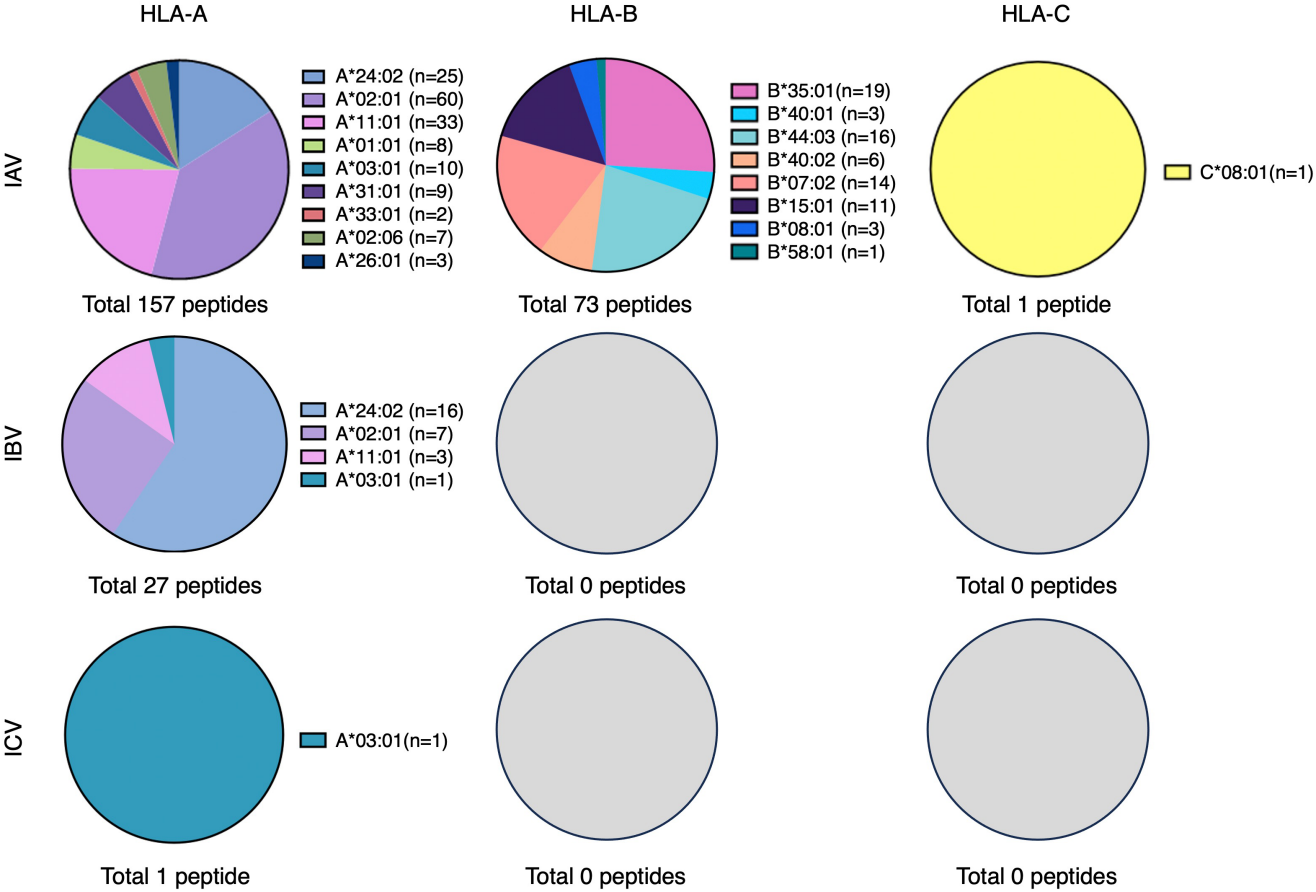
Top 10 most prevalent HLA‐A, B and C molecules and their known influenza‐derived epitopes. Known immunogenic IAV‐, IBV‐ and ICV‐derived epitopes restricted by the top 10 HLA‐A, B and C molecules were gathered from the Immune Epitope database in March and October 2023 (https://www.iedb.org/).^100^ The defined search result was restricted to linear peptides only, the influenza A virus, human hosts, positive T‐cell‐based assays only and the HLA‐I molecule of interest. Summary of the frequency of published epitopes per HLA molecule.

Subsequently, we reviewed how many known IAV‐, IBV‐ and ICV‐derived epitopes have been reported for the top 10 most prevalent HLA‐A, ‐B and ‐C molecules using the immune epitope database (IEDB)^100^ (Figure 1, Table 4 and Supplementary table 1). We limited the search results to positive T‐cell responses as indicative of epitopes. These positive responses were identified using a range of assays including intracellular cytokine staining, ELISPOT, tetramer staining and 51 chromium release assays. For our analysis, epitopes with an identical sequence and the same HLA‐I restriction were considered a single epitope. Epitopes with an identical sequence but different HLA‐I restriction are considered separate epitopes. Note that these epitopes are of variable immunogenicity, ranging from highly to weakly immunogenic. Since future vaccines should predominately include highly immunogenic epitopes, it would be important to consider the minimal epitope and level of immunogenicity of these epitopes, which is outside of the scope of this review, before considering their potential as vaccine candidates.

**Table 4:** Number of published T‐cell epitopes for the top 10 HLA‐A, ‐B and ‐C molecules

HLA‐A	Number of epitopes				Number of epitopes				Number of epitopes		
	IAV	IBV	ICV	HLA‐B	IAV	IBV	ICV	HLA‐C	IAV	IBV	ICV
A*24:02	25	16	0	B*35:01	19	0	0	C*07:02	0	0	0
A*02:01	60	7	0	B*51:01	0	0	0	C*04:01	0	0	0
A*11:01	33	3	0	B*40:01	3	0	0	C*03:04	0	0	0
A*01:01	8	0	0	B*44:03	16	0	0	C*01:02	0	0	0
A*03:01	10	1	1	B*40:02	6	0	0	C*07:01	0	0	0
A*31:01	9	0	0	B*07:02	14	0	0	C*06:02	0	0	0
A*33:03	2	0	0	B*15:01	11	0	0	C*03:03	0	0	0
A*02:06	7	0	0	B*07:04	0	0	0	C*08:01	1	0	0
A*26:01	3	0	0	B*08:01	3	0	0	C*15:02	0	0	0
A*30:01	0	0	0	B*58:01	1	0	0	C*12:02	0	0	0

Top 10 HLA‐A, B and C molecules were gathered from the Immune Epitope database (https://www.iedb.org/) in March and October 2023.^100^ The defined search result was restricted to linear peptides, influenza A, B or C virus (separately), human hosts, positive T‐cell‐based assays only and the HLA‐I molecule of interest. Note that some epitopes were restricted to more than one HLA molecule, as described by their respective publications.

Interestingly, the current landscape of known IAV‐derived T‐cell epitopes is highly focused on only 4 of the top 30 HLA‐I molecules (HLA‐A*24:02, ‐A*02:01, ‐A*11:01 and ‐B*35:01), with ~60% of the IAV‐derived epitopes identified binding to one of these 4 HLA‐I molecules (*n* = 137/231). From the 231 IAV‐derived epitopes assessed, 157 were restricted to HLA‐A molecules (68%), 73 to HLA‐B molecules (32%) and one to HLA‐C (< 0.1%) (Figure 1, Table 4). The HLA molecule with the most known epitopes is HLA‐A*02:01 (*n* = 60 epitopes), which is the second most prevalent HLA‐A molecule with a global expression of 15.2% (Figure 1, Tables 3 and 4). Other HLA molecules with similar (HLA‐A*11:01 at 11.6%, HLA‐C*04:01 at 11.17%) or higher (HLA‐A*24:01 at 18.80%) global prevalence, have fewer to no known IAV‐derived epitopes (Tables 3 and 4).

Like IAV, the majority (16/27 or 60%) of the IBV epitopes are restricted to a single HLA‐I molecule, namely HLA‐A*24:02 (Figure 1, Table 4). In contrast to IAV, HLA‐A*02:01 exclusively has seven IBV epitopes published, while HLA‐A*11:01 and HLA‐A*03:01 have a combined total of four peptides published (HLA‐A*11:01; *n* = 3, ‐A*03:01; *n* = 1). No IBV epitopes have so far been published for HLA‐B or HLA‐C. Strikingly, only a single weakly immunogenic ICV‐derived epitope has been reported according to the IEDB and is restricted to HLA‐A*03:01 from a single paper published in 2022.^3^

Together, this analysis suggests that most HLA‐I molecules have no known influenza‐derived epitopes, making them significantly understudied. Thus, these HLA‐I molecules should be a focus for future epitope identification studies to prove new knowledge and permit the selection of the best epitopes for inclusion in future influenza vaccines to provide broad population coverage.

## Current landscape of known epitopes and their proteins

**Figure 2:**
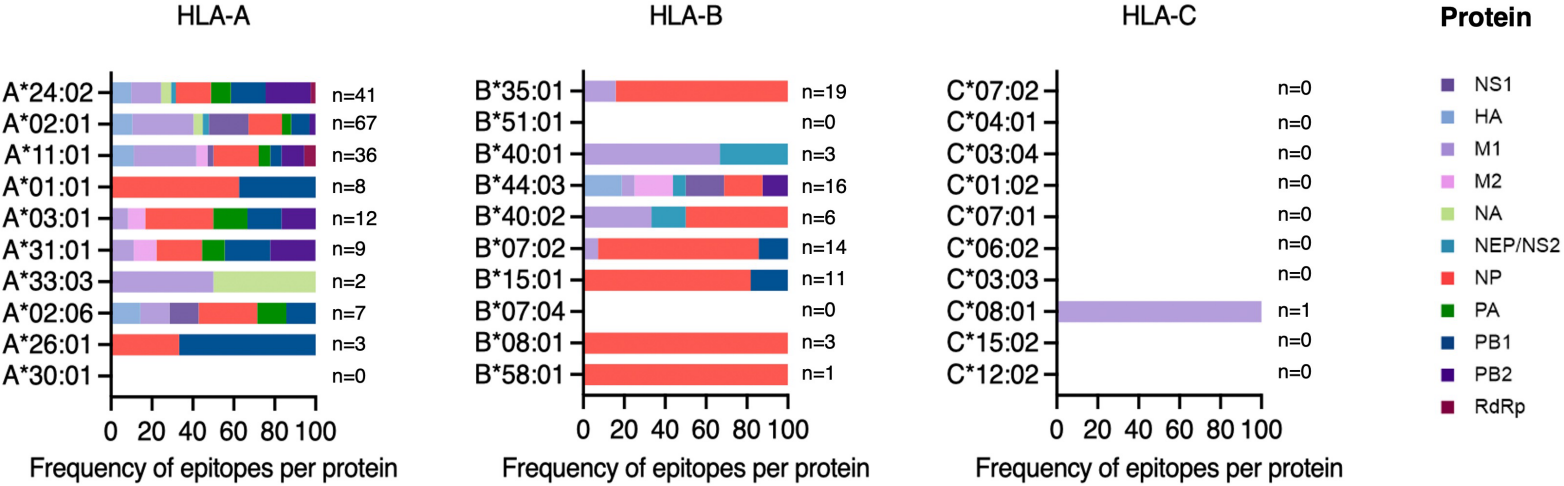
Top 10 most prevalent HLA‐A, ‐B and ‐C‐restricted influenza‐derived epitopes and their protein of origin. Access to the immune epitope database https://www.iedb.org/ ^100^ was used to identify influenza‐derived epitopes (IAV, IBV and ICV) restricted to the top 10 most prevalent HLA‐A, ‐B and ‐C‐restricted peptides and their protein of origin as per this figure. Data are shown as stacked bar graphs representing the frequency of epitopes per influenza protein, where *n* is the number of epitopes published for each HLA‐A, ‐B and ‐C molecule.

**Figure 3:**
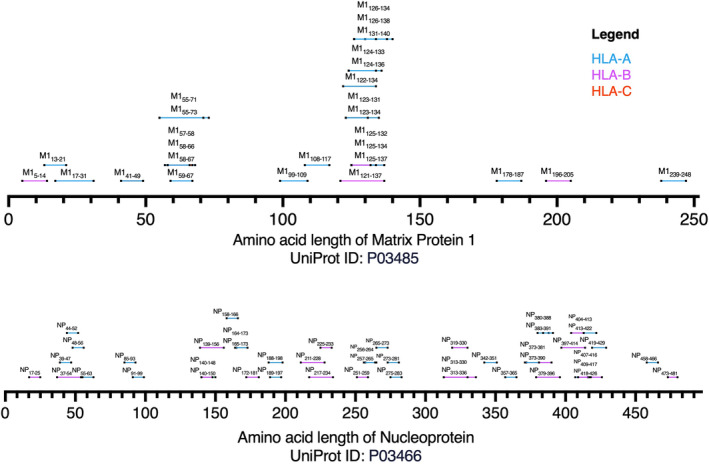
Epitope mapping of known immunogenic IAV‐, IBV‐ and ICV‐derived epitopes restricted by the top 10 HLA‐A, ‐B and ‐C molecules to NP and M1. NP‐ and M1‐derived immunogenic epitopes were mapped to their respective protein to visualise the cluster of sequences along a given protein. Each protein sequence length is directly proportional to the A/Puerto Rico/8/1934 H1N1 influenza A strain (UniProt ID: P03485 and P03466), downloaded from https://www.uniprot.org/.^105^ Each line represents a specific peptide sequence, name and length. The green line represents epitopes restricted to HLA‐A, pink for HLA‐B and red for HLA‐C. All sequences that contained a single amino acid change were included as the same epitope distance/length. NP_511–520_ was not included as the epitope position extended past the A/Puerto Rico/8/1934 H1N1 protein sequence (P03466).

Future influenza vaccines could induce CD8^+^ T‐cell responses by including individual epitopes, overlapping epitopes or conserved epitope‐rich regions of different influenza proteins. As such, we briefly looked at the known influenza‐derived epitopes, despite being focused on select HLA‐I molecules, to see where they mapped against the influenza proteins (Figures 2 and 3).

The known immunogenic IAV‐derived epitopes originated from a range of influenza proteins (Figure 2). Of note, epitopes derived from the nucleoprotein (NP) and matrix 1 (M1) were well represented across the HLA‐A and HLA‐B molecules, and the single IAV‐derived epitope restricted to the HLA‐C molecule was also M1 derived. For IBV and ICV, most epitopes were also derived from NP and M1 proteins, with a small proportion of HA and NS1 proteins observed for HLA‐A*02:01. The NP‐ and M1‐derived epitopes were also spread across the entire length of their respective proteins, with several sets of overlapping epitopes, some of which are likely to contain shared core epitopes (Figure 3).

These data collectively suggest that M1 and NP contain most of the known epitopes so far. This is consistent with reports of NP and M1 being a major target of CD8^+^ T‐cell responses across donors with distinct HLA‐I profiles.^88^, ^103^ Importantly, these proteins are known to be conserved, and a set of IAV‐derived epitopes, from NP and M1 were deemed ‘universally conserved’ across several IAV strains including an avian‐derived H7N9 strain.^85^ It will be interesting to find out whether these proteins also contain novel epitopes restricted to the understudied, yet prevalent HLA molecules, which might make them ideal proteins for future influenza vaccines. Indeed, Muraduzzaman *et al*.^16^ recently also eluted to the idea of targeting NP‐ and M1‐derived epitopes for universal CD8^+^ T‐cell flu vaccine.

## Conclusion

The influenza virus continues to cause severe respiratory illnesses and mortalities annually and remains a threat to human health with the potential to be responsible for future global pandemics. Although the CDC, TGA and the European medicines agency have approved influenza vaccines for administration, their efficacies fluctuate yearly depending on the circulating strains. Predicting which influenza strains will be circulating 6 months in the future can lead to mismatching vaccines with poorer efficacies. Thus, if a global pandemic were to arise, current strategies may not suffice. Fortunately, as time progresses, new vaccine technologies are made available. Currently, the main clinical trial vaccines for the influenza virus fall into one of several technologies including genetically modified influenza vaccine, VLP vaccines, nanoparticle vaccines, viral vector vaccines, mRNA vaccines or recombinant protein‐based vaccines. Many of these vaccines induce a neutralising antibody response to the surface glycoproteins. However, a select few vaccines also induce a T‐cell‐mediated response that may offer better and longer lasting protection. CD8^+^ T cells typically recognise peptides of more conserved protein origins. However, HLA‐I polymorphism poses a significant challenge in designing CD8^+^ T‐cell targets for inclusion in future vaccines.

In this review, we assessed the known immunogenic epitopes for the most prevalent HLA‐A, ‐B and ‐C molecules worldwide, showing that most HLA‐I molecules have no known influenza‐derived epitopes, with the majority of those published focused on a select few well‐studied HLA molecules. Thus, the rationale for future vaccine design is imperative to fill this gap in knowledge and identify novel epitopes restricted to these understudied but prevalent HLA‐I molecules.

## Conflict of interest

The authors declare no conflict of interest.

## Author contributions

**Samuel Liwei Leong:** Data curation; formal analysis; funding acquisition; investigation; project administration; writing – original draft; writing – review and editing. **Stephanie Gras:** Conceptualization; formal analysis; funding acquisition; project administration; supervision; writing – review and editing. **Emma J Grant:** Conceptualization; formal analysis; funding acquisition; project administration; supervision; validation; writing – original draft; writing – review and editing.

## Supporting information


Supplementary table 1
